# The Severe Acute Respiratory Syndrome Coronavirus-2 (SARS CoV-2) in Dentistry. Management of Biological Risk in Dental Practice

**DOI:** 10.3390/ijerph17093067

**Published:** 2020-04-28

**Authors:** Roberto Lo Giudice

**Affiliations:** Department of Clinical and Experimental Medicine, Messina University, 98123 Messina, Italy; rlogiudice@unime.it; Tel.: +39-393-439-9197

**Keywords:** COVID-19, SARS-CoV-2, dentistry, biological risk, infection prevention, procedures

## Abstract

The Severe Acute Respiratory Syndrome Coronavirus 2 (SARS-CoV-2) is a novel coronavirus first identified in Wuhan, China, and the etiological agent of Coronavirus Disease-2019 (COVID-19). This infection spreads mainly through direct contact with Flügge micro droplets or core droplets that remain suspended as aerosol. Moreover, it has been reported that infected subjects, both with and without clinical signs of COVID-19, can transmit the virus. Since the infection typically enters through mouth, nose, and eyes, dentistry is one of the medical practices at highest risk of infection due to the frequent production of aerosol and the constant presence of saliva. The World Health Organization (WHO) has suggested that only emergency/urgent procedures should be performed during the coronavirus outbreak. Considering the virus’ route of transmission, a specific protocol should be applied to reduce the risk of infection in addition to measures that prevent the spread of infection from a patient to another person or medical tools and equipment (cross-infection). This protocol should be implemented by modifying both patient management and clinical practice, introducing particular devices and organizational practices. This paper aims to discuss and suggest the most appropriate procedures in every aspect of dental practice to reduce infection risk.

## 1. Introduction

The Severe Air Respiratory Syndrome (SARS) Coronavirus 2 (SARS-CoV-2) is a single-stranded RNA virus (+ssRNA) of 60–140 nm, belonging to the β-Coronavirus genus (subgenus sarbecovirus, Orthocoronavirinae subfamily), and the etiological agent of Coronavirus Disease-2019 (COVID-19). The virus has a crown-like appearance due to the presence of spike glycoproteins on the envelope, and is considerably different genetically from SARS-CoV and MERS-CoV [1,2].

### 1.1. Epidemiology

The outbreak of SARS-CoV-2 was first reported on 12 December 2019 in Wuhan, China, possibly related to a seafood market [2].

Based on data retrieved from the World Health Organization (WHO), at the date of the present article (22 April 2020), there are 2,471,136 confirmed cases, with 169,006 deaths, and the virus is present in 213 countries, areas, or territories [3].

The median age of patients is 47–59 years and 41.9%–45.7% of patients are female [2].

Due to the widespread and rapid growth of infection, WHO declared that the global outbreak of the new coronavirus infection SARS-CoV-2 can be considered a pandemic [3].

### 1.2. Route of Transmission

Genetic and epidemiologic research shows that the COVID-19 outbreak likely started through animal-to-human transmission, followed by human-to-human diffusion [2].

The coronavirus exploits angiotensin-converting enzyme 2 receptor (ACE2), which is found in the lower respiratory tract. This is similar to the route of infection in humans for SARS-CoV, which mainly spreads through the respiratory tract [2].

The virus is transmitted through Flügge micro droplets (droplets) and core droplets (aerosol) [1].

Diffusion mainly occurs through coughing, sneezing, and saliva. The distance and length of time that particles remain suspended in the air is determined by particle size, settling velocity, relative humidity, and air flow [1].

**Figure 1:**
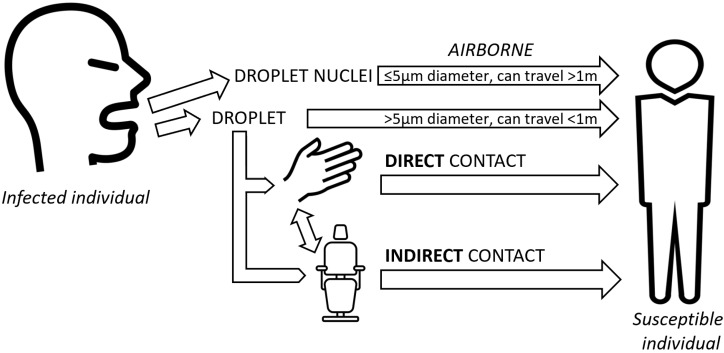
Severe Acute Respiratory Syndrome Coronavirus 2 (SARS-CoV-2) route of transmission of in healthcare settings.

Flügge droplets that are >5 µm in diameter can spread up to 1 m. The nuclei of the droplets which have a diameter <5 µm, create an aerosol which has a diffusion capacity greater than 1 m (Figure 1) [4].

Infection entry points are the mouth, nose, and eyes. In rare cases, the virus can be transmitted by the oro-fecal route [1,4].

The propagation of infected droplets occurs through contact with infected subjects, with or without clinical signs of COVID-19. Many observations have reported that even asymptomatic patients in the incubation phase or healthy carriers can transmit the virus [5,6].

Infection can occur as a result of close direct interpersonal proximity (distance less than 2 m and duration greater than 15 min. or following contact with hands which have come into contact with contaminated surfaces or airborne particles [4].

SARS-CoV-2 could present an asymptomatic incubation period for infected individuals that varies from 5 or 6 to 14 days. Backer has reported that virus identification in human respiratory epithelial cells could be positive about 96 h from exposure and 24−48 h before the onset of symptoms [7,8].

The most frequent symptoms are: body temperatures >37.4 °C, dry cough, dyspnea, asthenia, muscle pain, headache, sore throat, diarrhea, and vomiting [9].

### 1.3. Clinical Classification

In relation to the symptoms, the disease can be classified as follows [10].

**Mild form**: Mild symptoms, no signs of pneumonia are observed at radiology.

**Moderate form**: Body temperature >37.4 °C, respiratory symptoms, signs of pneumonia are observed with radiology.

**Severe and very severe form**: Usually occurs seven days after the infection. Dyspnea and/or hypoxemia may occur in patients with severe forms of the disease, with rapid progression to Acute Respiratory Distress Syndrome (ARDS), septic shock, and acidosis. In critical/very severe patients, severe metabolic alterations, coagulation deficiency, and multiple organ failure can occur. The rise in body temperature in these patients may be mild or absent.

### 1.4. Differential Diagnosis

The symptoms of the mild form of COVID-19 are nonspecific. Differential diagnosis should be made using a wide range of infectious (e.g., adenovirus, influenza, human metapneumovirus (HmPV), parainfluenza, respiratory syncytial virus (RSV), rhinovirus (common cold)) and non-infectious (e.g., vasculitis, dermatomyositis, organized cryptogenetic pneumonia) common respiratory disorders. 

Rapid antigen detection in the nasal and throat cavity or other respiratory tracts is currently the best clinical diagnosis method of COVID-19. This method and other investigations should be performed to differentiate COVID-19 from common respiratory pathogens and other non-infectious conditions [2,11]. Scientific knowledge on its clinical evolution is constantly being updated.

## 2. Infection Risk in Dental Practice

Biological risk is an intrinsic threat in dental practice, to which patients, doctors, assistants, hygienists, and all other staff may be exposed.

Medical activities carried out in dental practice must always refer to procedures for the assessment and prevention of risks posed by the potential transmission of an infectious biological agent.

However, the procedures adopted routinely to date have not been specifically designed for the prevention of pathogens transmissible by aerosol. Therefore, there are currently no specific guidelines for the protection of dentists against SARS-CoV-2 [3].

Due to the transmission route, in addition to measures that prevent diffusion of the infection from a patient to another person or medical tools and equipment (cross-infection), it is advisable to add further airborne and contact precautions to the routine standard hygienic procedures in order to reduce the risk of SARS-CoV-2 transmission.

In a dental practice, the prevention, control, and reduction of infection transmission risk commonly takes place through:

- The use of personal protective equipment (PPE) such as gloves, masks, visors, goggles, dental uniform, and surgical gown and shoes (see section on PPEs below).
- A set of decontamination, disinfection, and sterilization procedures aimed at inactivating, destroying, or removing pathogens from any surface or instruments [12].

Prevention of SARS-CoV-2 infection must consider its spread through air and the size of droplets (<5 µm or >5 µm). The disease prevention measures should also take into account the potential ability of the virus to contaminate surfaces [4].

Although certain data are not available, the WHO reports that virus persistence on surfaces can vary from a few hours to a few days in relation to environmental parameters and the contaminated surface [13]. An environment with low relative humidity is reported to decrease the persistence of SARS-CoV-2 [13].

The SARS-CoV-2 virus is sensitive to ultraviolet rays and heat, and can be inactivated at a temperature of 56 °C for 30 min, as well as by lipid solvents such as ether, 75% ethanol, and disinfectants containing chlorine, peracetic acid, and chloroform. It is not sensitive to chlorhexidine [13].

The aim of this article is to focus on hygienic procedures within the dental practice during a coronavirus pandemic.

## 3. Hygiene Measures among Dental Practice Professionals

### 3.1. Generic Measures

**Figure 2:**
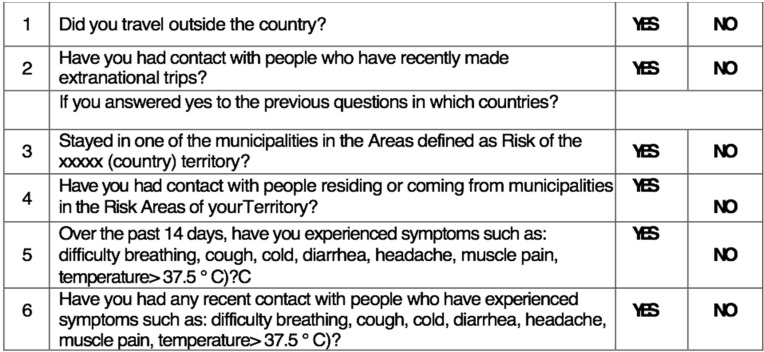
Additional questions to the medical record for SARS-CoV-2 (12 March 2020).

To reduce the risk of SARS-CoV-2 infection, given how the disease spreads and the current health crisis, the following prevention measures are suggested in addition to what is already generally performed:

- Inform patients of the procedures for accessing the dental office.
- Inform the patient that a telephone triage will be administered and any suspicious case will be reported to competent health authorities for further investigation.
- Carry out a preliminary telephone triage upon patient access to the office in order to assess whether:

1. The patient who is entering the office has symptoms of SARS-CoV-2 infection;
2. Has come into contact with potentially infected people;
3. Has been in areas with high risk of infection (Figure 2).

In the event of a positive response, do not schedule appointments. Instead, inform the patient of the possible risk of being infected, the personal hygienic measures they need to follow, and that they will be reported to the competent health authorities for further investigations.

**Figure 3:**
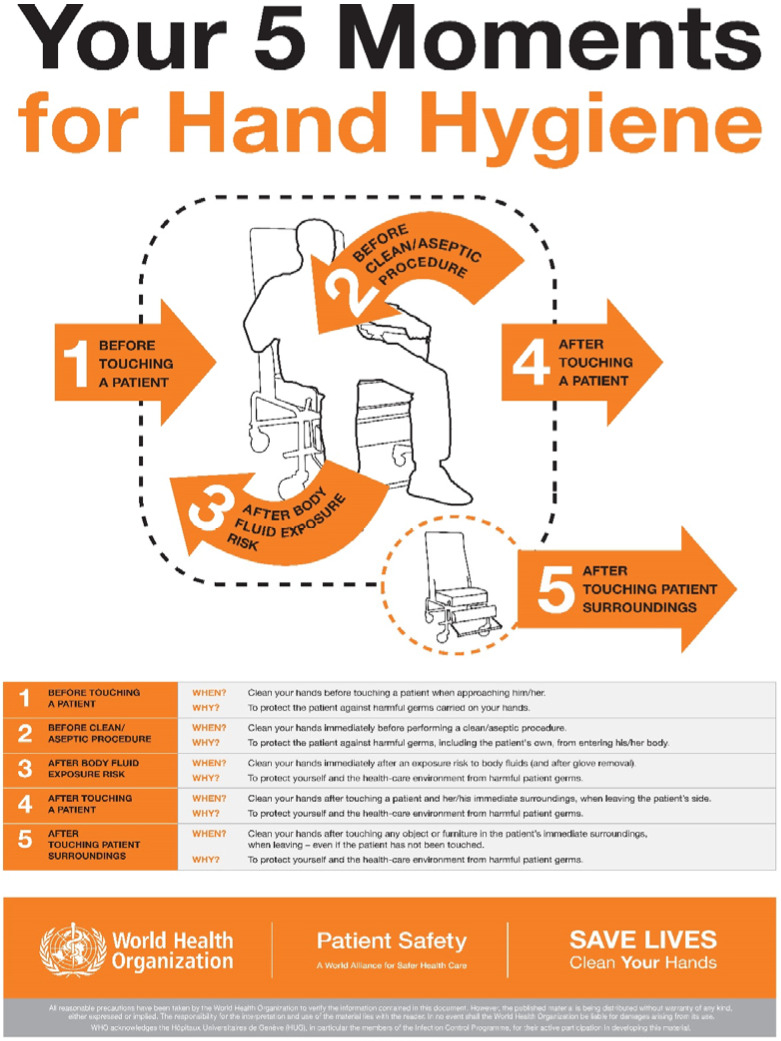
Moments For Hand Hygiene [from WHO Clean care is safer care Your 5 Moments for Hand Hygiene https://www.who.int/gpsc/5may/tools/workplace_reminders/en/ Revised August 2009 (Accessed on 24 March 2020)].

**Figure 4:**
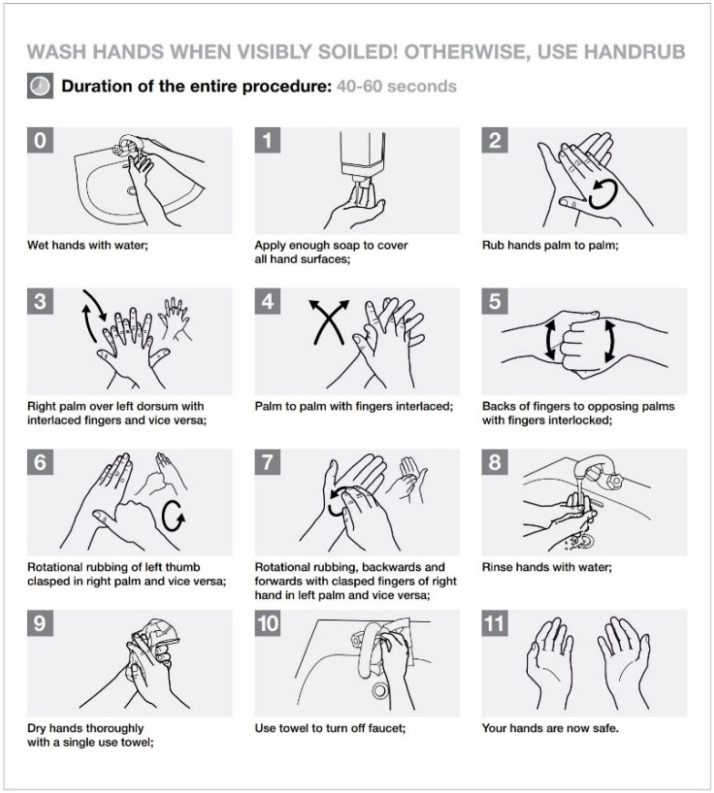
How to Hand Wash? [From: WHO Patient safety. Hand Hygiene: Why, How & When? Revised August 2009 https://www.who.int/gpsc/5may/tools/workplace_reminders/en/ (Accessed on 24 March 2020)].

**Figure 5:**
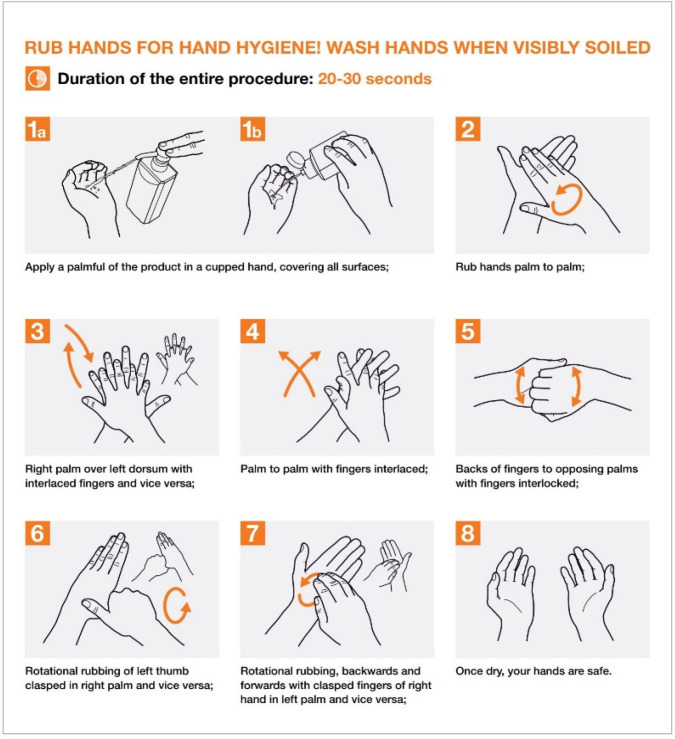
How to Hand Rub? [From: WHO Patient safety. Hand Hygiene: Why, How & When? Revised August 2009 https://www.who.int/gpsc/5may/tools/workplace_reminders/en/ (Accessed on 24 March 2020)].

- Only schedule an appointment in the dental office if the patient cannot be deferred or in urgent cases and according to the equipment and disposable materials available.
- Assess the actual need for dental intervention before planning visits of the most vulnerable subjects (elderly or patients with respiratory, cardiovascular, or immune system diseases, etc.). Where appropriate, postpone visits [14].
- Avoid crowding by spreading appointments over time, maintaining distance in the waiting room and/or in the operating rooms (minimum safety distance of 2 m with a stay of less than 15 min) [4].
- Arrange entry into the operating rooms of individual patients and a single companion for minors, without overcoats, electronic devices, and bags, which must be left in the waiting room. In the event that personal effects should enter the operating rooms, these objects must be placed in special sealed bags.
- Ensure that hand sanitizer is available to patients and companions, possibly at the entrance of the practice or anywhere before entering the operating rooms.
- In the patient’s medical history, record the results of the telephone triage regarding the presence of suspicious and actual symptoms, potential contact with people who have been to risk areas, and the patient’s own transit/permanence in risk areas (Figure 2).
- Remind the patient that, in case of necessity, it is mandatory to cough by covering their mouth and nose, possibly with a tissue. The tissue must be disposed of immediately in a special waste container. Following this, they must proceed to wash and disinfect hands.
- Always allow fresh air in between one patient and another, and frequently in the waiting room. This action could be performed by opening the windows, taking care of the air influx, or using medical-grade air purifiers as recommended by the manufacturer.
- Consider all material that comes into direct or indirect contact with the patient and all material that has been used for patient treatment or has come in contact with any biological fluid as special waste. Manage it according to the proper disposal techniques to ensure that any infectious materials cannot contaminate or spread to other areas.
- Always use PPE. The PPE should be used as described by the instructions contained in the user manual and must be disposed of as special waste. Always check the integrity of the PPE, and, in the event of an integrity breach, eliminate PPE directly.
- Use a medical cap.
- Scrupulously observe hand hygiene (Figure 3, Figure 4 and Figure 5).

Before performing hand hygiene procedures, it is suggested to:

1. Expose forearms (bare below the elbows);
2. Remove all hand and wrist jewelry;
3. Ensure fingernails are clean and short. Artificial nails or nail products are not recommended;
4. Cover all cuts or abrasions with a waterproof dressing.

- Remove potentially contaminating objects from waiting rooms and operating rooms (magazines, etc.).
- Regularly sanitize common and operational areas, non-medical furnishings and equipment, and surfaces accessible to the public (handles, etc.).
- Place protections on points of sale (POSs), keyboards, etc. and change them after each use. A single-use protection could be a disposable barrier (plastic film or cover) that must be replaced after every use.
- Arrange the use of respiratory protective devices (RPDs) for all staff including secretarial staff (see section on PPEs below).
- Inform doctors and collaborators concerning the clinical triage for COVID-19 and train them to evaluate themselves. If they have manifested symptoms of a coronavirus infection (cough, cold, diarrhea, flu symptoms, temperature >37.5 °C) in the last 14 days, they should not come into the dental office and should report themselves to the competent health authorities [9].
- Suggest intensifying personal hygiene procedures at the end of the work shift.

### 3.2. Specific Measures 

Considering that each of the various dental disciplines has its operating peculiarities, it is appropriate to distinguish between particular precautions as follows.

#### 3.2.1. Precautions in Operative Areas

The following procedures should be adopted:

- Protect the surfaces of all equipment and instruments with single-use disposable barriers, and dispose of the protections among the special waste after use.
- Arrange only strictly necessary material on the surfaces of operating areas.
- Clean the operative surfaces with hydroalcoholic disinfectants at concentrations above 60%.
- Wear a uniform with long sleeves and shoes, and avoid exposed body parts.
- Wear a disposable lab coat/overcoat.
- It is also suggested that the patient mouth-rinse for 30 s with a 1% solution of hydrogen peroxide (1 part at 10-volume/3% hydrogen peroxide and 2 parts of water) or with 1% iodopovidone. This pre-operative treatment could lower the virus concentration in the patient’s mouth [3].
- Give preference to extra-oral radiological examinations over intra-oral ones to avoid the stimulation of coughing or vomiting [15].

#### 3.2.2. Precautions from Procedures that Produce Droplets or Aerosol

The following procedures should be adopted in addition to the ones described above:

- Replace and sterilize the high- and low-speed hand-pieces after each use between one patient and the next; the use of 3-way turbines and dental units equipped with valves and anti-reflux systems is recommended [8].
- Use RPDs and protective glasses for eye protection or full visors for all medical and paramedical staff in contact with the patient. If not available, surgical masks with full visors can be used for all medical and paramedical staff in contact with the patient [3,16] (see section on PPEs below).
- When possible, apply a rubber dam to reduce possible aerosol production [17,18].

## 4. Personal Protective Equipment 

Wearing PPE provides a physical barrier that could prevent contact between medical and paramedical staff and the pathogen, as well as potentially infected biological material.

They must be worn and replaced at the end of each operating phase and disposed of in the special waste.

All protective devices must be replaced as soon as they are damaged or before loss of effectiveness.

### 4.1. Respiratory Protective Devices

Respiratory protective devices are commonly used to protect wearers from chemical, biological, and radioactive materials.

These devices have been classified by the US National Institute for Occupational Safety and Health (NIOSH), which sorts particulate filtering face-piece respirators (FFRs) into nine categories (N95, N99, N100, P95, P99, P100, R95, R99, and R100) [19].

The European Standard (EN 149:2001) classifies FFRs into three classes, FFP1, FFP2, and FFP3, with minimum respective filtration efficiencies of 80%, 94%, and 99% of the particles with a dimension up to 0.6 μm. An FFP2 is comparable to a N95 FFR [20].

The various indications of how to use FFRs is related to their different capacity and quality of filtration.

Surgical masks (SMs) can filter particles of 0.04–1.3 μm, and are commonly used to physically block particles such as droplets. Their principal limitation is due to poor quality of face fit and the consequent possibility of aerosol aspiration. The use of SMs protects the patient from saliva and respiratory secretion produced by healthcare workers.

The FFP1 and FFP2 masks are available with or without an exhalation valve, and FFP3 masks always have a valve. The FFP2 and FFP3 masks are the most appropriate barriers against aerosol because they provide a tight seal to the facial skin.

Considering that the air flow is filtered in the inhalation phase, but not filtered during exhalation (which is expelled from the valve), the infection risk is moved from operator to patient.

Masks with exhale valves are therefore not recommended for use in dentistry as this will increase the patient’s risk if the professional is infected with the virus, especially considering that the latter could be asymptomatic.

#### 4.1.1. Indications

- Wear a surgical mask in all eventualities where there is contact between the patient and other people less than 2 m away and for more than 15 min.
- Wear an FFP2 or FFP3 mask in procedures with a risk of aerosol droplet production. If not available, use a surgical mask with a face shield [3].
- Remove the mask after each aerosol risk procedure by touching only the strings.

#### 4.1.2. Common Mistakes

- Removing the mask by touching the possibly contaminated surface. The correct procedure is as follows: After removing the gloves, put on a new pair of gloves, and remove the mask by the strings.

### 4.2. Gloves

#### 4.2.1. Indications

**Figure 6:**
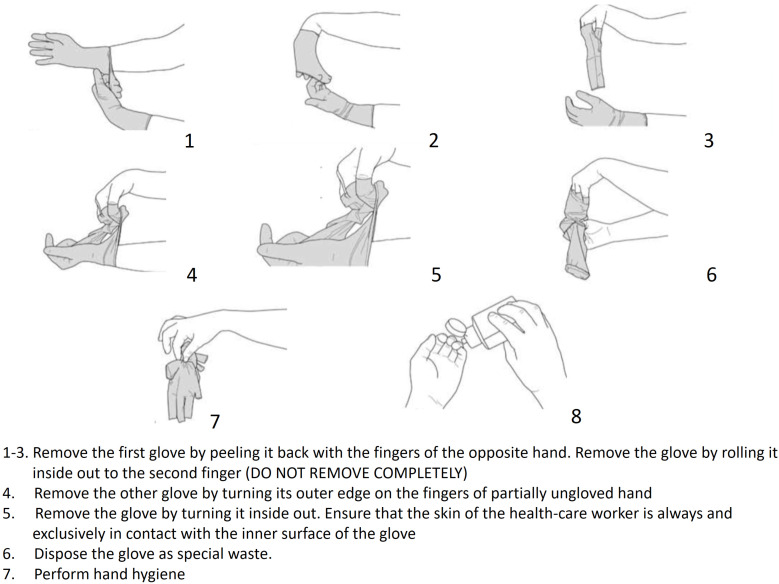
How to remove gloves.

- Disposable gloves must be used in all operating phases that involve contact with potentially infected surfaces and for direct patient assistance.
- Gloves should be inspected before use.
- A double pair of gloves can serve as additional protection.
- Before putting on gloves, cover any cuts or damaged portions of hand skin as an additional precaution.
- After use, gloves should be removed using appropriate techniques to prevent hand contamination. Gloves should be removed by rolling from the wrist towards the fingers to avoid contact with skin. It is necessary to remove gloves by touching only the external part (Figure 6).
- Use sterile gloves for invasive procedures that require surgical asepsis.

#### 4.2.2. Common Mistakes

- Using the same pair of gloves for different operations and/or for different patients.
- Not washing hands before and after using gloves.

### 4.3. Face and Eye Protection 

#### 4.3.1. Indications

- Eyes should be protected with goggles or a full-face shield in all procedures at risk of aerosol/droplet production.
- Glasses, visors, or face shields can be used.
- After every operation, depending on the kind of procedure, protections must be disposed of or sterilized.

#### 4.3.2. Common Mistakes

- Only using common prescription glasses.
- Only using medical loupes.
- Removing protections after each procedure by touching the external surface. Strings must be used for removal.

### 4.4. Dental Uniform/Surgical Gown 

#### 4.4.1. Indications

- Always use a uniform with long sleeves and shoes.
- Change the uniform every day.
- Before washing, disinfect with a chlorine solution (500 mg/L) for 30 s [18].
- Wear a surgical gown (over the uniform) for each procedure at risk of contamination with aerosol/droplets.
- Dispose of the surgical gown after each visit. Remove the surgical gown before glove removal and touch it only from the outside.
- Remove the surgical gown by folding it with the potentially contaminated external surface inside.
- In case of contamination, replace the dental uniform as soon as possible.

#### 4.4.2. Common Mistakes

- Applying standard precautions when handling and replacing clothing.
- Replacing the surgical gown after glove removal.
- Removing the dental uniform without a new pair of gloves.

### 4.5. Recommended Sequence of PPE Removal

1. Remove any overcoat.
2. Remove the gloves by rolling them up from the wrist, without touching the skin.
3. Wash hands.
4. Wear a new pair of gloves to avoid accidental contact.
5. Remove the protective goggles or visor and mask, taking care to touch only the strings and not the contaminated surface.
6. Remove the gloves.
7. Wash hands.

## 5. Discussion

The pandemic infection of SARS-CoV-2 could have a profound impact on dentistry, mainly due to the way the pathogen is passed on, which poses a danger in almost all dental procedures. The virus predominantly spreads through droplets and aerosol, requiring a revision of the cross-infection prevention protocol to include SARS-CoV-2 related risks [3].

Especially when treating in urgent situations or emergencies during the virus pandemic [6], clinicians should not underestimate the possibility of infection from asymptomatic patients, and all dental treatment should be considered high-risk. The dentist should always use the recommended personal protective equipment, including filtering face-piece respirators that could prevent aerosol infection and protective glasses or face shields that could prevent droplets from coming into contact with the eye mucosa.

The management of patients should include a proper pre-treatment telephonic triage to screen out patients at risk of contagion and follow an appropriate schedule to avoid crowding.

## 6. Conclusions

In this paper, the most appropriate procedures were discussed and suggested to minimize the risk of infection in every aspect of dental practice.

## References

[1] Peng X., Xu X., Li Y., Cheng L., Zhou X., Ren B. (2020). Transmission routes of 2019-nCoV and controls in dental practice. Int. J. Oral Sci..

[2] Guo Y.-R., Cao Q.-D., Hong Z.-S., Tan Y.-Y., Chen S.-D., Jin H.-J., Tan K.-S., Wang D.-Y., Yan Y. (2020). The origin, transmission and clinical therapies on coronavirus disease 2019 (COVID-19) outbreak—An update on the status. Mil. Med. Res..

[3] WHO.int. 2020. Coronavirus Situation Report-83. https://www.who.int/emergencies/diseases/novel-coronavirus-2019/situation-reports/&gt;.

[4] Otter J.A., Donskey C., Yezli S., Douthwaite S., Goldenberg S.D., Weber D.J. (2016). Transmission of SARS and MERS coronaviruses and influenza virus in healthcare settings: The possible role of dry surface contamination. J. Hosp. Infect..

[5] Chan J.F.-W., Yuan S., Kok K.-H., To K.K.-W., Chu H., Yang J., Xing F., BNurs J.L., Yip C.C.-Y., Poon R.W.-S. (2020). A familial cluster of pneumonia associated with the 2019 novel coronavirus indicating person-to-person transmission: A study of a family cluster. Lancet.

[6] Rothe C., Schunk M., Sothmann P., Bretzel G., Froeschl G., Wallrauch C., Zimmer T., Thiel V., Janke C., Guggemos W. (2020). Transmission of 2019-NCOV infection from an asymptomatic contact in Germany. N. Engl. J. Med..

[7] Backer J.A., Klinkenberg D., Wallinga J. (2020). Incubation period of 2019 novel coronavirus (2019-nCoV) infections among travellers from Wuhan, China, 20–28 January. Eurosurveill.

[8] Meng L., Hua F., Bian Z. (2020). Coronavirus Disease 2019 (COVID-19): Emerging and Future Challenges for Dental and Oral Medicine. J. Dent. Res..

[9] Chen N., Zhou M., Dong X., Qu J., Gong F., Han Y., Qiu Y., Wang J., Liu Y., Wei Y. (2020). Epidemiological and clinical characteristics of 99 cases of 2019 novel coronavirus pneumonia in Wuhan, China: A descriptive study. Lancet.

[10] National Health Commission of the People’s Republic of China (2020). Chinese Clinical Guidance for COVID-19 Pneumonia Diagnosis and Treatment.

[11] Cascella M., Rajnik M., Cuomo A., Dulebohn S.C., Di Napoli R. (2020). Features, evaluation and treatment coronavirus (COVID-19). Treasure Island (FL).

[12] Spagnuolo G., De Vito D., Rengo S., Tatullo M. (2020). COVID-19 outbreak: An overview on dentistry. Int. J. Environ. Res. Public Health.

[13] Xu K., Lai X., Liu Z. (2020). Suggestions on the prevention of COVID-19 for health care workers in department of otorhinolaryngology head and neck surgery. World J. Otorhinolaryngol. Head Neck Surg..

[14] Cascella M., Rajnik M., Cuomo A., Dulebohn S.C., Di Napoli R. (2020). Features, Evaluation and Treatment Coronavirus (COVID-19).

[15] Vandenberghe B., Jacobs R., Bosmans H. (2010). Modern dental imaging: A review of the current technology and clinical applications in dental practice. Eur. Radiol..

[16] ECDC TECHNICAL REPORT Infection Prevention and Control for COVID-19 in Healthcare Settings March. https://www.ecdc.europa.eu/en/publications-data/infection-prevention-and-control-covid-19-healthcare-settings.

[17] Samaranayake L.P., Reid J., Evans D. (1989). The efficacy of rubber dam isolation in reducing atmospheric bacterial contamination. ASDC J. Dent. Child..

[18] Li Z.Y., Meng L.Y. (2020). The prevention and control of a new coronavirus infection in department of stomatology. Chin. J. Stomatol..

[19] National Institute for Occupational Safety and Hygiene (NIOSH) (1996). NIOSH Guide to the Selection and Use of Particulate Respirators Certified under 42 CFR 84.

[20] Lee S.-A., Hwang D.-C., Li H.-Y., Tsai C.-F., Chen C.-W., Chen J.-K. (2016). Particle size-selective assessment of protection of european standard FFP respirators and surgical masks against particles-tested with human subjects. J. Healthc. Eng..

